# Immunomodulatory and Antioxidant Activities of a Polysaccharide from *Ligustrum vicaryi* L. Fruit

**DOI:** 10.1155/2020/5431350

**Published:** 2020-03-17

**Authors:** Shuling Liu, Litao Wang, Qiang Ren, Jianan Wang, Yanzhi Li, Guanghui Wang, Huijie Gao, Rui Du, Wei Qin

**Affiliations:** School of Pharmacy, Jining Medical University, Rizhao 276800, China

## Abstract

*Ligustrum vicaryi* L. is a hybrid of *Ligustrum ovalifolium* Hassk. var. *aureo-marginatum* and *Ligustrum vulgale* L., belonging to the Oleaceae family. It is often used as an ornamental shrub due to its golden leaves. However, its medical value is yet to be discovered. Recently, plant polysaccharides have attracted comprehensive attention owing to their biological properties, including immunomodulatory and antioxidant activities. This study aimed to extract, purify, and characterize the polysaccharide from the *Ligustrum vicaryi* L. fruit and investigate its immunomodulatory and antioxidant activities. The *Ligustrum vicaryi* L. fruit polysaccharide (LVFP) was obtained by ultrasonic extraction, ethanol precipitation, macroporous resin separation, and dialysis bag purification. The physicochemical properties of the LVFP were elucidated using Fourier-transform infrared spectrometry, high-performance ion chromatography, and high-performance gel filtration chromatography. The results indicated that the LVFP consisted of rhamnose, arabinose, galactose, and glucose in a ratio of 1.79 : 7.55 : 4.58 : 1.54, and its molecular weight was 88,949 Da. The immunomodulatory and antioxidant activities of the LVFP were investigated using a cyclophosphamide- (Cy-) induced immunosuppressed mouse model. The results demonstrated that the LVFP significantly increased spleen and thymus indexes, enhanced the phagocytic function of neutrophils, activated B and T lymphocytes, and upregulated serum levels of IL-10 and TNF-*α*. Moreover, we observed that the LVFP relieved Cy-induced liver damage by increasing superoxide dismutase (SOD) and glutathione peroxidase (GSH-px) levels. These results suggested that the LVFP has the immunomodulatory and antioxidant activities, therefore laying a foundation for the application of the LVFP in the pharmaceutical and functional food industries.

## 1. Introduction

Immunomodulators can be used as preventive or therapeutic strategies, boosting or suppressing the host defense response. Natural products with immunomodulatory and antioxidant functions are widely used to treat several diseases, including autoimmune disease, inflammatory disorder, and cancer [1, 2]. Recently, there has been growing interest in inexpensive and less toxic natural products over the use of synthetic chemotherapeutic agents.

*Ligustrum vicaryi* L. is a hybrid of *Ligustrum ovalifolium* Hassk. var. *aureo-marginatum* and *Ligustrum vulgale* L., belonging to the Oleaceae family [3]. It presents a chlorophyll-less phenotype and is widely used as a horticultural shrub owing to its golden leaves. According to the gas-exchange characteristics and chlorophyll fluorescence responses, *Ligustrum vicaryi* L. can resist SO_2_ and thus can be used for the phytostabilization of Cd-contaminated soil [4, 5]. Although *Ligustrum vicaryi* L. has high ornamental and environmental protection value, its therapeutic potential is yet to be elucidated.

Plant polysaccharides, a class of important biological macromolecules commonly found in traditional medicinal herbs, exhibit a range of biological activities including antioxidant, immunomodulatory, antiaging, antitumor, and anti-inflammatory activities [6–8]. In recent years, there have been many studies dedicated to the extraction and bioactivity analysis of plant polysaccharides. Plant fruit polysaccharides are the most commonly researched, including the jujube polysaccharides [9], persimmon polysaccharides [10], and watermelon polysaccharides [11]. To the best of our knowledge, a study based on the preparation and bioactivities of *Ligustrum vicaryi* L. fruit polysaccharides (LVFPs) has not been reported.

In this study, the LVFP was extracted and purified. Next, the molecular weight and monosaccharide composition of the LVFP were analyzed. Moreover, the biological functions of the LVFP were assessed in a cyclophosphamide- (Cy-) induced immunosuppressed mouse model. The results demonstrated that the LVFP has immunomodulatory ability by activating both innate and adaptive immunity. Furthermore, the LVFP demonstrated antioxidant activity by increasing superoxide dismutase (SOD) and glutathione peroxidase (GSH-px). This study presented a theoretical basis for developing the LVFP as an alternative therapeutic remedy for immune-related diseases.

## 2. Materials and Methods

### 2.1. Animal Welfare and Ethical Statements

All experimental procedures complied with the recommendations of ARRIVE (animal research: reporting *in vivo* experiments) guidelines [12]. The Institutional Animal Care and Use Committee of Jining Medical University approved the procedures. All experimental procedures were conducted as humanely as possible. A total of 100 healthy male Kunming mice weighing 20–25 g were housed at conditions maintaining 12 h of a dark-light artificial cycle, with food and water available *ad libitum*. The animals were housed in standard animal rooms, with a temperature of 20–22°C and humidity at 55–60%.

### 2.2. Extraction and Purification of Polysaccharide

*Ligustrum vicaryi* L. fruits were obtained from the botanical garden of Jining Medical University (Rizhao, Shandong, China) and identified by Jianan Wang (Medical Botanist, School of Pharmacy, Jining Medical University). Dried *Ligustrum vicaryi* L. fruits were pulverized and sifted through a 40-mesh sieve. The powder was immersed in acetone (the ratio of powder to acetone was 1 : 3) and shaken for 24 h to remove lipids. The supernatant was discarded, and the leftover was dried in the oven. The polysaccharide was extracted with distilled water 8 : 1 (v/w) at 50°C for 80 min using an ultrasonic power of 250 W. The water extract was filtered using cotton. The residue was removed after centrifugation at 3000 rpm for 3 min. Next, the concentrated supernatant was precipitated with 70% ethanol, and this process was repeated with 95% ethanol. After the supernatant was removed, distilled water was added to dissolve the extract, and the residue was removed by centrifugation. The DM101 macroporous resin was used to remove the pigment. The Sevage reagent (chloroform/1-butanol, v/v = 4 : 1) was used to remove the protein until no discoloration reaction was observed using Coomassie Brilliant Blue G-250 detection. The polysaccharides were placed in a freezing dryer for 24 h to obtain the frozen powder. The small molecules in the polysaccharide were removed by using a dialysis bag (molecular cutoff of 3500 Da) for two days, and distilled water was replaced every 12 h. Column chromatography Sephadex G-200 was used to purify the LVFP. The anthrone-sulfuric acid assay, with a glucose standard curve, was used to determine the purity of the LVFP.

### 2.3. Fourier-Transform Infrared (FTIR) Analysis

The LVFP was mixed with pure potassium bromide powder, and the mixture was placed in an agate mortar and ground uniformly under an infrared lamp. The mixture was assessed using the Fourier-transform infrared spectrometer IRTracer-100 (Shimadzu, Japan).

### 2.4. High-Performance Ion Chromatography (HPIC)

For HPIC analysis, 5 mg LVFP was dissolved in 1 mL of 2 mol/L trifluoroacetic acid and placed at 121°C for 2 h for hydrolysis. Then, the mixture was filtered using a 0.45 *μ*m microporous filtration membrane. The chromatographic separation of monosaccharides was performed on the ICS-5000 ion chromatograph equipped with a CarboPac PA20 column and pulse ampere detector. The ion chromatography separation conditions were adjusted and tested for a mixture of eight monosaccharide standards (fucose, rhamnose, arabinose, galactose, glucose, xylose, mannose, and fructose). A superior separation was obtained by gradient elution with a constant flow rate of 0.5 mL/min. The mobile phase consisted of 250 mM NaOH (A), water (containing 1 M NaAc%, v/v, B), and water (C). The elution was carried out as follows: 2.0% A at 0∼21 min; 2.0% A and 5.0% B at 21.1 min; 2.0% A and 20% B at 21.1∼30 min; and 80% A at 30.1∼50 min.

### 2.5. High-Performance Gel Filtration Chromatography (HPGFC)

The polysaccharide molecular mass was analyzed by HPGFC (Waters, New York, USA) and equipped with the RID (2414 refractive index detector) and Empower 3 workstation. The chromatographic conditions were as follows: chromatographic column: Ultrahydrogel™ Linear, 300 mm × 7.8 mm × 2, mobile phase: 0.1 M NaNO_3_, flow rate: 0.9 mL/min, and column temperature: 45°C. The sample was dissolved in the liquid phase and filtered by a microporous filtration membrane. Molecular weight standards used for the calibration curve were MW135350, MW36800, MW9750, MW2700, and MW180.

### 2.6. Cyclophosphamide- (Cy-) Induced Immunosuppressive Mouse Model and LVFP Administration

A total of 100 mice were randomly divided into five groups: control, Cy, Cy + LVFP (100 mg/kg/day), Cy + LVFP (200 mg/kg/day), and Cy + LVFP (400 mg/kg/day). The control group was administered normal saline, and the other four groups were intraperitoneally injected with Cy (40 mg/kg) every day for seven days. The LVFP was administered by intragastric administration for seven consecutive days.

### 2.7. Determination of Spleen and Thymus Indexes

All mice were weighed and sacrificed 24 h after the last drug administration. The spleen and thymus were excised and weighed. The spleen or thymus index was expressed as the ratio of the spleen or thymus weight to body weight.

### 2.8. Determination of the Phagocytic Function of Mouse Neutrophils

*Staphylococcus aureus* was used to assess the phagocytosis of neutrophils. In brief, 40 *μ*L of blood was obtained from mice after LVFP administration and placed in a heparin tube to prevent coagulation. Next, 40 *μ*L of bacterial suspension was added to the above-mentioned tube. The mixture was evenly coated on glass slides and maintained at room temperature for 0.5 h. Then, the mixture was fixed with 2-3 drops of methanol for 5 min and stained with 4-5 drops of Wright's stain for 5 min. A total of 100 neutrophils were observed and recorded. The neutrophil phagocytic rate and the neutrophil phagocytic index were calculated as follows: the neutrophil phagocytic rate = the number of neutrophils that displayed phagocytic function/the total number of neutrophils and the neutrophil phagocytic index = the total number of bacteria which had been engulfed by neutrophils/the total number of neutrophils.

### 2.9. Determination of Serum Hemolysin by ELISA

On the fourth day, 5% of a chicken red blood cell (CRBC) suspension was intraperitoneally injected into the mice (0.1 mL/10 g). On the seventh day, 2 h after LVFP administration, 1 mL of mice blood was obtained and centrifuged for 10 min. Then, 40 *μ*L of serum was added to 2 mL normal saline. One mL 10% guinea pig serum and 1 mL 5% CRBC suspension were added to the serum, followed by incubation in a water bath at 37°C for 0.5 h. The mixture was centrifuged, and the supernatant was placed in a 96-well culture plate. The corresponding optical density value was detected by the Thermo Scientific Multiskan MK_3_ Enzyme Mark instrument (Waltham, MA, USA).

### 2.10. Determination of T Lymphocyte Transformation Rate

On the second day, the mice were intramuscularly injected 8 mg/kg of phytohemagglutinin (PHA). On the seventh day, 2 h after LVFP administration, 40 *μ*L of blood was collected from the retroorbital sinus, placed on a slide, and stained with 4-5 drops of Wright's stain. The excess dye was rinsed with water after 20 min. Using a microscope, 100 cells were observed. The transformed lymphocytes were calculated as follows: lymphocyte transformation rate = transformed lymphocytes/total number of lymphocytes.

### 2.11. Delayed-Type Hypersensitivity (DTH) Response

DTH response was examined by the degree of ear swelling. Generally, the degree of ear swelling is believed to reflect the degree of inflammation [13]. DTH response was measured as follows: On the second day, 1 cm^2^ of abdominal hair was removed using Na_2_S and the abdominal skin was covered with 25 *μ*L of 1-fluoro-2,4-dinitrobenzene (DNFB) solution. On the sixth day, the skin on the right ear was coated with 20 *μ*L of DNFB solution. On the seventh day, 2 h after the LVFP administration, the mice were sacrificed and the two ears were excised and weighed. The weight difference between the two ears determined the degree of ear swelling.

### 2.12. Detection of TNF-*α* and IL-10 Cytokines in Serum

On the seventh day, 2 h after LVFP administration, the mice blood was obtained using a retroorbital sinus puncture and naturally coagulated for 15 min at room temperature. Next, the blood was centrifuged for 20 min, and the supernatant was collected. TNF-*α* and IL-10 in the supernatant were detected using ELISA kits (Nanjing Jiancheng Bioengineering Institute, Nanjing, China).

### 2.13. Detection of Superoxide Dismutase (SOD), Glutathione Peroxidase (GSH-Px), and Methane Dicarboxylic Aldehyde (MDA) Levels

On the eighth day, the mice were sacrificed and the livers were extracted. After grinding and centrifugation, the supernatant of liver tissue was collected for the detection of SOD, GSH-px, and MDA. SOD, GSH-px, and MDA were measured using ELISA kits (Nanjing Jiancheng Bioengineering Institute, Nanjing, China).

### 2.14. Hematoxylin and Eosin (HE) Staining

HE staining was performed as previously reported [14]. On the eighth day, the mice were sacrificed and liver tissues were extracted and fixed in 10% neutral-buffered formalin. After embedding in paraffin, the tissues were cut into 5 *μ*m sections. Then, the slides were stained with HE to determine morphological changes. Images were photographed under a light microscope (Olympus, Tokyo, Japan).

### 2.15. Statistical Analysis

Experimental subjects/preparations were randomly assigned to groups, and equal group sizes were obtained. Group assignments, data recording, and data analysis were not disclosed to the investigator. Data are shown as the mean ± SD from at least five independent experiments. One-way ANOVA accompanied by Tukey's multiple-comparisons test was used for multiple comparisons using GraphPad Prism version 6.0 (GraphPad Software, La Jolla, CA, USA) [15], and *p* < 0.05 was considered statistically significant.

## 3. Results

### 3.1. FTIR Spectrum Analysis of LVFP

**Figure 1:**
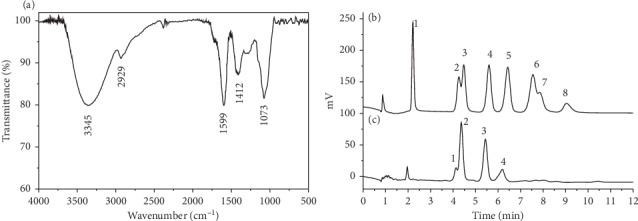
Structural characterization of the *Ligustrum vicaryi* L. fruit polysaccharide (LVFP). (a) Fourier-transform infrared spectrum analysis of the LVFP. (b) High-performance ion chromatography analysis of monosaccharide standards. 1, fucose; 2, rhamnose; 3, arabinose; 4, galactose; 5, glucose; 6, xylose; 7, mannose; 8, fructose. (c) High-performance ion chromatography analysis of the LVFP. 1, rhamnose; 2, arabinose; 3, galactose; 4, glucose.

The LVFP was extracted by lipid removal via acetone, hot water combined with ultrasound extraction, ethanol precipitation, pigment removal by macroporous resin, deproteination with the Sevage reagent, and small molecule removal by a dialysis bag, respectively. Moreover, the novel polysaccharide structure was characterized by infrared spectroscopy. The FTIR spectrum analysis of the LVFP is shown in Figure 1(a). The characteristic broad peak at 3345 cm^−1^ corresponded to the O-H (perhaps including N-H) stretching vibration. The absorption peak at 2929 cm^−1^ corresponded to the C-H stretching vibration absorption peak and sugar absorption peak. The absorption at 1599 cm^−1^ indicated the bending vibration modes of -OH. The peak at 1412 cm^−1^ resulted from the presence of C-H bending vibration. Furthermore, the absorptions at 1073 cm^−1^ indicated the bending vibration modes of C-O stretching in the alcoholic hydroxyl group form.

### 3.2. Monosaccharide Composition of LVFP

The monosaccharide compositions of the LVFP were analyzed by HPIC. As shown in Figures 1(b) and 1(c), all the monosaccharides existing in polysaccharides were identified according to the elution time of the monosaccharide standards, with reference to a standard curve. The LVFP was mainly composed of rhamnose, arabinose, galactose, and glucose in the molar ratio of 1.79 : 7.55 : 4.58 : 1.54.

### 3.3. Determination of Molecular Weight of LVFP by HPGFC

The molecular weight of the LVFP was detected by HPGFC. Dextrans (molecular weight: 180, 2700, 9750, 368000, and 135350 Da) were used as standards since they are water-soluble and available in a wide range of molecular masses. The standards provided guidelines on estimating the size of the LVFP. A standard curve was obtained (*y* = −0.523*x* + 13.01, *R*^2^ = 0.995). The results demonstrated that the molecular weight of the LVFP was 88,949 Da.

### 3.4. Effects of LVFP on Thymus and Spleen Indexes in Cy-Induced Immunosuppressive Mice

**Figure 2:**
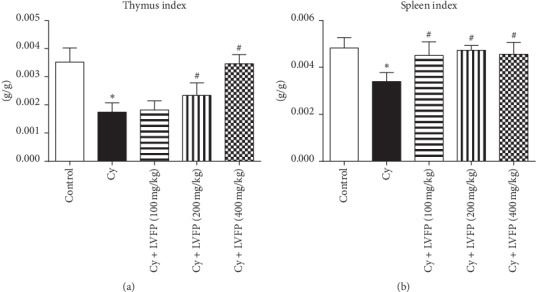
Effects of the *Ligustrum vicaryi* L. fruit polysaccharide (LVFP) on thymus and spleen indexes in cyclophosphamide- (Cy-) induced immunosuppressive mice. (a) LVFP increases the thymus index. *n* = 7–10. (b) LVFP increases the spleen index. *n* = 7–10. Data are shown as the mean ± SD; ^*∗*^*p* < 0.05*vs.* the control group; ^#^*p* < 0.05*vs.* the Cy group.

The Cy-induced immunosuppressed mouse model was established to investigate the immunomodulatory activity of the LVFP. Cy was administered as an intraperitoneal injection (40 mg/kg) every day for seven days. After five days of Cy administration, the mice exhibited symptoms such as hair loss, low excitement, aggression, poor appetite, and weight loss, whereas no obvious change was observed in the control group, indicating that the animal model was successfully established. Cy + LVFP groups were administrated different concentrations of the LVFP (100, 200, and 400 mg/kg/day) for seven consecutive days. Spleen and thymus indexes reflect the immune functions of the organism [16]. The LVFP effects on the thymus and spleen indexes in the Cy-induced immunosuppressive mice are shown in Figure 2. Compared to the control group, the Cy group displayed a significant decrease in thymus and spleen indexes. LVFP administration significantly increased these indexes in a dose-dependent manner. These results suggested that the LVFP could affect the immune organs to enhance immunity.

### 3.5. Effects of LVFP on Neutrophil Phagocytosis in Cy-Induced Immunosuppressive Mice

**Figure 3:**
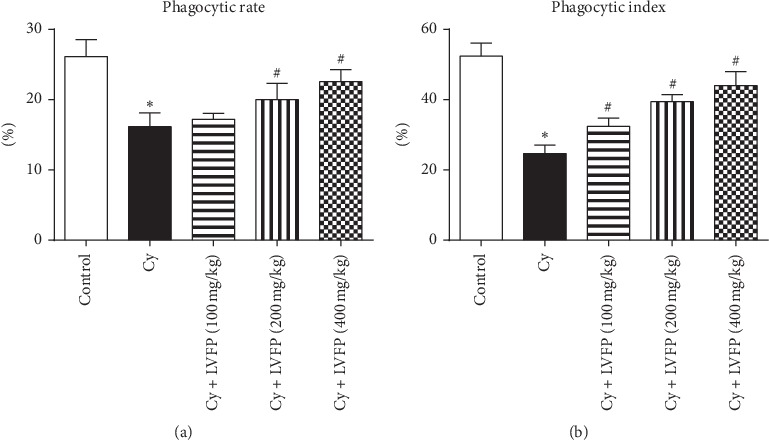
Effects of the *Ligustrum vicaryi* L. fruit polysaccharide (LVFP) on the phagocytic rate and phagocytic index in cyclophosphamide- (Cy-) induced immunosuppressive mice. (a) LVFP increases the neutrophil phagocytic rate. *n* = 5–8. (b) LVFP increases the neutrophil phagocytic index. *n* = 5–8. Data are shown as the mean ± SD; ^*∗*^*p* < 0.05*vs.* the control group; ^#^*p* < 0.05*vs*. the Cy group.

Neutrophils are an effective defense against invading microorganisms [17]. Phagocytosis is a key strategy for neutrophils in killing pathogens [18]. The phagocytic rate and phagocytic index were examined to investigate the activation of neutrophils [19]. As shown in Figure 3, compared with the control group, the Cy group had significantly reduced phagocytic rate and phagocytic index, suggesting an immunosuppressive status. LVFP (200 mg/kg and 400 mg/kg) administration significantly alleviated these changes. These results suggested that the LVFP can enhance the phagocytic function of neutrophils.

### 3.6. Effects of LVFP on Humoral Immunity and Cellular Immunity in Cy-Induced Immunosuppressive Mice

**Figure 4:**
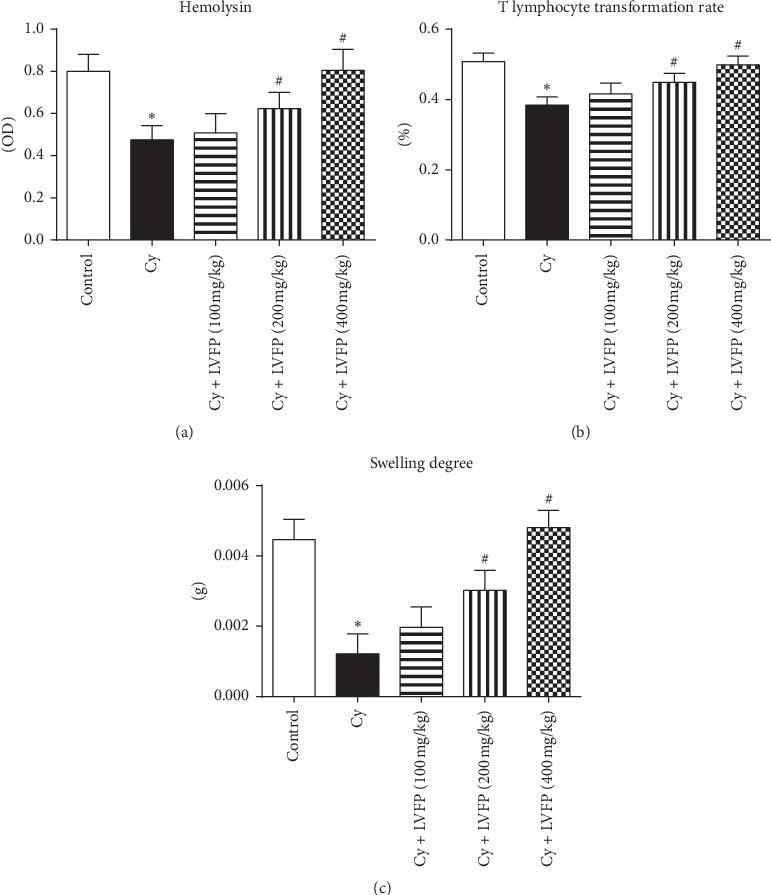
Effects of the *Ligustrum vicaryi* L. fruit polysaccharide (LVFP) on hemolysin, T lymphocyte transformation rate, and delayed-type hypersensitivity response in cyclophosphamide- (Cy-) induced immunosuppressive mice. (a) LVFP increases the serum hemolysin level. *n* = 7–10. (b) LVFP increases the T lymphocyte transformation rate. *n* = 9–10. (c) LVFP increases the ear swelling degree. *n* = 5–9. Data are shown as the mean ± SD; ^*∗*^*p* < 0.05*vs.* the control group; ^#^*p* < 0.05*vs*. the Cy group.

Humoral immunity is critical for the body to fight tumors and infections. The serum hemolysin level can be used to evaluate the humoral immune response [20]. The serum hemolysin was decreased by Cy treatment, and this change was attenuated significantly by the LVFP in a dose-dependent manner (Figure 4(a)). A variety of antigens from blood can activate T lymphocytes. PHA can act as a mitogen to trigger the activation and proliferation of T cells [21]. The T lymphocyte transformation assay was performed using mouse blood after PHA stimulation in the presence or absence of the LVFP. As shown in Figure 4(b), all three doses of the LVFP markedly inhibited the Cy-induced reduction of the T lymphocyte transformation rate. The DTH response is a T cell-mediated immune response [22]. The DTH response was assessed by measuring the degree of ear swelling. All three doses of the LVFP markedly attenuated Cy-induced inhibition in the ear swelling degree (Figure 4(c)). These results suggested that the LVFP can activate B and T lymphocytes.

### 3.7. Effects of LVFP on the Expression of IL-10 and TNF-*α* in the Serum of Cy-Induced Immunosuppressive Mice

**Figure 5:**
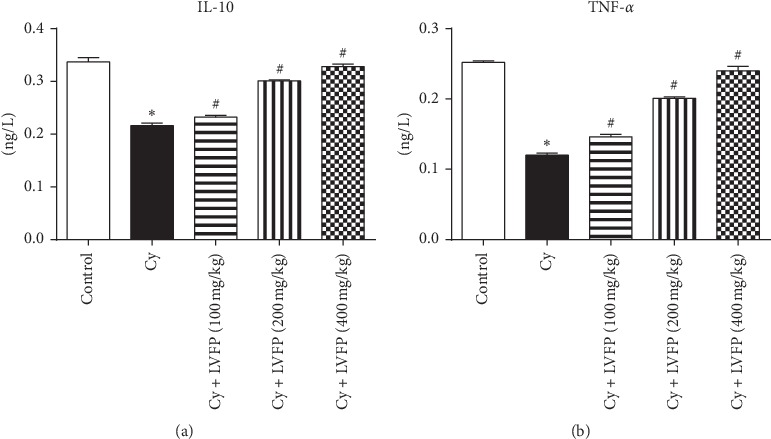
Effects of the *Ligustrum vicaryi* L. fruit polysaccharide (LVFP) on IL-10 and TNF-*α* expressions in cyclophosphamide- (Cy-) induced immunosuppressive mice. (a) LVFP increases the serum IL-10 expression. *n* = 8. (b) LVFP increases the serum TNF-*α* expression. *n* = 8. Data are shown as the mean ± SD; ^*∗*^*p* < 0.05*vs*. the control group; ^#^*p* < 0.05*vs*. the Cy group.

Cytokines secreted by immune cells play vital roles in host defense [23]. IL-10 and TNF-*α* are key inflammatory mediators [24]. ELISA was performed to ascertain the effects of the LVFP on the serum expression of IL-10 and TNF-*α*. Our results demonstrated that the IL-10 and TNF-*α* expressions, after Cy treatment, were significantly decreased. LVFP administration dose-dependently increased IL-10 and TNF-*α* levels (Figure 5).

### 3.8. Effects of LVFP on Oxidative Stress in Cy-Induced Immunosuppressive Mice

**Figure 6:**
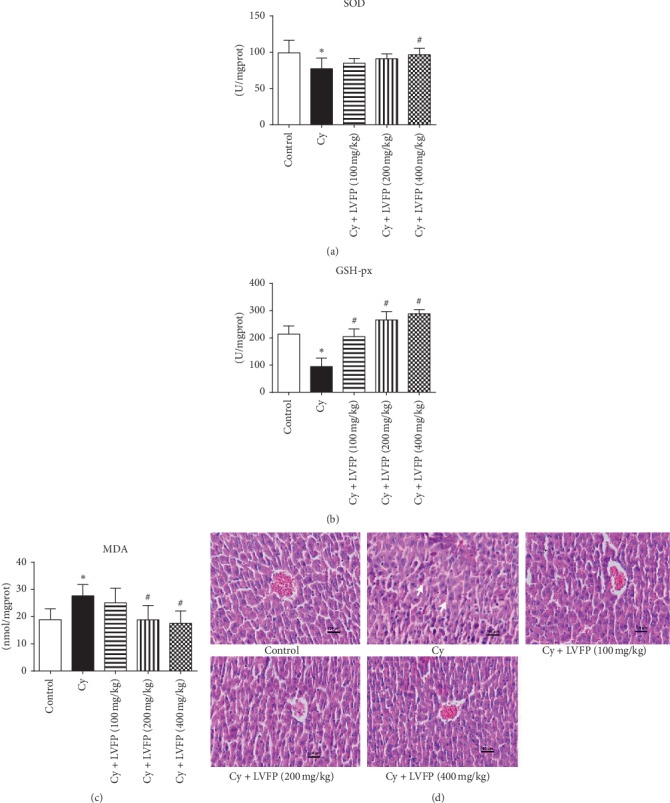
Protective effects of the *Ligustrum vicaryi* L. fruit polysaccharide (LVFP) on liver injury induced by cyclophosphamide (Cy). Effects of the LVFP on liver SOD (a), GSH-px (b), and MDA (c) levels in immunosuppressed mice. *n* = 5–7. (d) Representative photographs of HE-stained sections of liver tissue. Arrows indicate that hepatic sinusoids are full of red blood cells. Scale bar indicates 100 *μ*m. Data are shown as the mean ± SD; ^*∗*^*p* < 0.05*vs*. control; ^#^*p* < 0.05*vs*. Cy.

Furthermore, this study investigated whether the LVFP relieved Cy-induced oxidative stress and liver damage. SOD converts the superoxide anion into H_2_O_2_ and O_2_ to prevent and neutralize the free radical-induced damage [25]. GSH-px is presumed to be an important endogenous defense against peroxidative destruction of the cellular membrane [26]. MDA is the end product of lipid peroxidation and can reflect the oxidative damage in the liver tissue [27]. Figures 6(a)–6(c) demonstrate the significantly decreased SOD and GSH-px expressions, with increased MDA levels in the liver tissue after Cy treatment. Administration of the LVFP dose-dependently increased SOD and GSH-px levels and decreased the MDA level. Next, the changes in liver morphology were assessed using HE staining. As shown in Figure 6(d), in the Cy group, the liver cells were sparse and disordered with the pyknotic nucleus. The hepatic sinusoids were full of red blood cells. As expected, the liver cells in the LVFP group were arranged in an orderly manner with a clear nucleolus, comparable to those in the control group. The results indicated that the LVFP could attenuate the oxidative stress and liver damage induced by Cy.

## 4. Discussion

In this study, a polysaccharide was extracted from the *Ligustrum vicaryi* L. fruit, and its immunomodulatory and antioxidant activities were assessed. The polysaccharide was extracted by hot water combined with ultrasound extraction, ethanol precipitation, fat removal by acetone, pigment removal by DM101 macroporous resin, deproteination with the Sevage reagent (chloroform/1-butanol, v/v = 4 : 1), and small molecule removal using the dialysis bag, respectively. Moreover, this polysaccharide structure was characterized by infrared spectroscopy, HPGFC-RID, and HPIC. The results indicated that the molecular weight of the LVFP is 88,949 Da, and the LVFP consists of four monosaccharides, namely, rhamnose, arabinose, galactose, and glucose in the molar ratio of 1.79 : 7.55 : 4.58 : 1.54.

Immunosuppression is a state of temporary or permanent immune dysfunction and can render an organism more sensitive to pathogens. Developing new immunomodulatory agents is one of the most effective methods for the prevention and treatment of immunosuppressive diseases [28]. For example, immunomodulatory agents combined with chemotherapy drugs appear to be useful in cancer therapy [29]. Several reports have indicated positive correlations between the immunomodulatory actions and polysaccharides. Ayeka et al. demonstrated that the licorice (*Glycyrrhiza uralensis* Fisch.) polysaccharide has immunomodulatory effects evident through the activation of CD4^+^ and CD8^+^ immune cell populations and increased production of various cytokines, such as IL-2, IL-6, and IL-7 [30]. Chen et al. isolated polysaccharides from *Schisandra sphenanthera* and *Schisandra chinensis*; these extracts could enhance the phagocytic activity of macrophages and improve the body's immunity [31]. Reportedly, it was observed that the polysaccharides from *Schisandra sphenanthera* and *Schisandra chinensis* were mainly composed of arabinose, glucose, and galactose, similar to the composition of the LVFP. To investigate whether the LVFP possessed immunomodulatory functions, a Cy-induced immunosuppressed mouse model was established. Cy is a chemotherapeutic agent and has been used to establish immunosuppressive animal models [32, 33]. At the level of immune organs, the results indicated that the LVFP significantly increased spleen and thymus indexes in Cy-induced immunosuppressive mice. At the level of immune cells, it was observed that the LVFP enhanced the phagocytic function of neutrophils and promoted B and T lymphocyte activation. These data indicate that the LVFP can enhance both innate and adaptive immunity.

Furthermore, immune-related cytokines in the serum were also examined, and the results indicated that the LVFP increased IL-10 and TNF-*α* levels. Reportedly, several types of polysaccharides can influence the expression of inflammatory factors. Chen et al. observed that sulfated polysaccharides from filamentous microalgae *Tribonema* sp. can enhance IL-6, IL-10, and TNF-*α* expressions in macrophages [34]. Cheng et al. reported that polysaccharides from the wild *Lactarius deliciosus* increased the secretion of TNF-*α*, IL-1*β*, and IL-6 in macrophages [35]. Wang et al. reported that the polysaccharide from the lichen *Umbilicaria esculenta* induced the release of TNF-*α*, IFN-*γ*, IL-1*β*, IL-6, and IL-10 in murine macrophages [36].

Antioxidants are substances that suppress oxidation in the human body. Usually, there is a balance between reactive oxygen species production and antioxidant defense system, which is indispensable in normal metabolism. Cells produce some endogenous antioxidants such as SOD and GSH-px to suppress excessive free radicals [37]. This study determined the antioxidant activity of the LVFP in Cy-induced immunosuppressive mice. The results demonstrated that the LVFP exhibited a significant increase in SOD and GSH-px expressions. Furthermore, the LVFP decreased MDA expression, which is indicative of oxidative damage in the liver. HE staining indicated that the LVFP can relieve liver cell damage induced by Cy. Thus, it can be concluded that the LVFP might protect cells from oxidative stress-induced damage by enhancing the production of antioxidants such as SOD and GSH-px. Previous research has shown that the content of rhamnose, arabinose, and galactose in the monosaccharide composition may affect the antioxidant activity [38, 39]. The content of rhamnose, arabinose, and galactose in the LVFP is higher, which may be important for the stronger antioxidant activity demonstrated by the LVFP.

Overall, polysaccharides from the *Ligustrum vicaryi* L. fruit were extracted and their physical and biological characteristics were investigated. The LVFP could be explored as a potential immunomodulatory and antioxidant agent for therapeutic use in immunosuppressive diseases, allowing for the introduction of traditional Chinese medicine to the international market.

## 5. Conclusions

**Figure 7:**
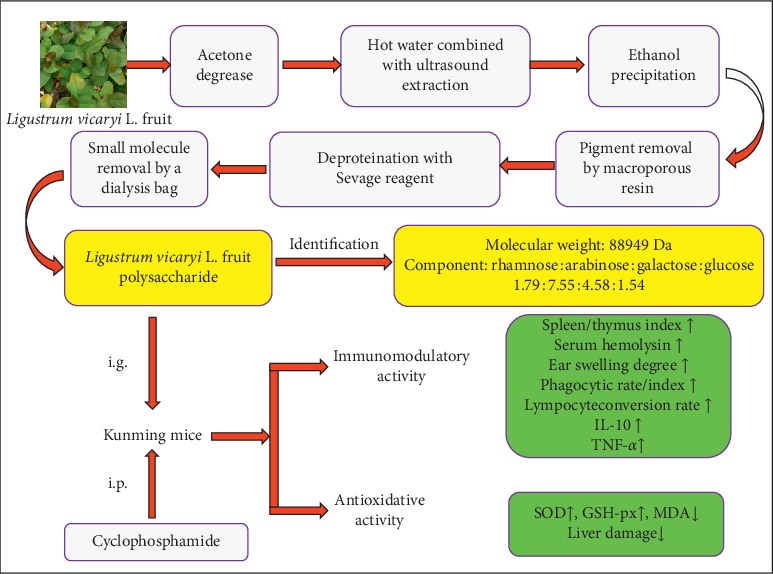
Flow chart of the extraction, structure characterization, and biological activity identification for the *Ligustrum vicaryi* L. fruit polysaccharide.

In conclusion, the LVFP was extracted and purified from the *Ligustrum vicaryi* L. fruit with a molecular weight of 88,949 Da and consisted of four monosaccharides, namely, rhamnose, arabinose, galactose, and glucose. Preliminary pharmacological studies indicated that the LVFP could effectively improve the immune functions and had antioxidant activity. This study demonstrates that the LVFP may be a promising immunomodulatory and antioxidant agent for pharmaceutical therapies (Figure 7).

## Data Availability

The TIF data used to support the findings of this study are available from the corresponding author upon request.
